# Infraoccluded permanent first molars and their effect on dentoalveolar and root development: a two-center retrospective split-mouth cross-sectional study

**DOI:** 10.1186/s40510-026-00650-1

**Published:** 2026-10-29

**Authors:** Avi Leibovich, Zehava Geller Fishman, Gabriel Nahmias, Heli Rushinek, Nardi Casap, Letizia Perillo, Miriam Shalish, Cristina Grippaudo, Ludovica Nucci, Stella Chaushu

**Affiliations:** 1 Department of Orthodontics, Hadassah Medical Center, Faculty of Dental Medicine, Hebrew University of Jerusalem, Jerusalem, Israel; 2 Faculty of Dental Medicine, Hebrew University of Jerusalem, Jerusalem, Israel; 3 Department of Oral and Maxillofacial Surgery, Hadassah Medical Center, Faculty of Dental Medicine, Hebrew University of Jerusalem, Jerusalem, Israel; 4 Multidisciplinary Department of Medical-Surgical and Dental Specialties, University of Campania Luigi Vanvitelli, Naples, Italy; 5 Dipartimento Universitario testa collo ed organi di senso, Università Cattolica del Sacro Cuore, 00168 Rome, Italy; 6 UOC Clinica Odontoiatrica, Dipartimento di Neuroscienze, Organi di senso e Torace, Fondazione Policlinico universitario A. Gemelli, IRCCS, 00168, Rome, Italy; 7 Department of Mental and Physical Health and Preventive Medicine, University of Campania “Luigi Vanvitelli”, Naples, Italy

**Keywords:** Infraocclusion, Ankylosis, Submergence, Root dilaceration

## Abstract

**Background:**

Infraocclusion of a permanent first molar, frequently due to ankylosis, can disrupt occlusal development and generate asymmetries. This study aimed to evaluate dental developmental differences caused by infraoccluded permanent first molars.

**Methodology:**

A retrospective, two-center, split-mouth cross-sectional study analyzed panoramic radiographs of 45 patients (9–25 years) with a single, unilateral, infraoccluded permanent first molar. The contralateral molar served as control. Measurements included infraocclusion severity, teeth position within the arch, adjacent-tooth tipping and spacing, root length, presence and degree of root dilaceration.

**Results:**

Infraoccluded permanent first molars were positioned 5.6(3.3) mm (95% CI, 5 to 7; *p* < 0.001) lower than controls and were significantly more distal within the arch (*p* < 0.001), with adjacent teeth also displaced distally. Adjacent teeth tipped toward the infraoccluded molar: second premolars were 8(12)° more distally angulated (95% CI, 5 to 12; *p* < 0.001) and second molars 6(19)° more mesially angulated (95% CI, 0.6 to 15; *p* = 0.003). Posterior spacing was more frequent on the affected side (OR = 15.5; 95% CI 4 to 133; *p* < 0.001). Infraoccluded molars had roots that were 2.8(5.4) mm shorter than controls (95% CI, 1.2 to 4.4; *p* < 0.001) and a markedly higher prevalence of root dilaceration (OR = 18.5; 95% CI 5 to 158; *p* < 0.001). Infraocclusion severity was linearly associated with dilaceration severity (*r* = 0.69; *p* = 0.0001). Adjacent teeth showed a similar trend toward higher prevalence and severity of dilacerations on the affected side.

**Conclusions:**

Unilateral infraoccluded permanent first molars are strongly associated with shortened roots, increased root dilaceration, and significant distal displacement of the affected and adjacent teeth. Other effects include tipping and spacing of the adjacent teeth.

## Introduction

Infraocclusion is a condition in which the occlusal surface of a tooth remains below the level of the adjacent teeth, persisting long after it should have reached its normal occlusal position. The exact etiology is unknown, however ankylosis is widely regarded as its primary cause [1, 2]. Histologically, ankylosis is the abnormal fusion of the root surface (either cementum or dentin) with the surrounding alveolar bone. Ankylosis can occur spontaneously, or due to environmental causes (trauma, local infection or invasive cervical root resorption). A genetic basis has also been suggested given the frequent association between infraocclusion and other dental anomalies [2–5]. Furthermore, infraocclusion due to ankylosis can happen at any stage throughout the eruption process, even after the tooth has reached the normal occlusal plane. As the affected tooth ceases to erupt, the surrounding tissues continue their vertical growth, resulting in progressive relative submergence, with clinical severity depending heavily on the timing of onset. Functional disturbances such as tongue thrusting or specialized bite habits can impede eruption and mimic relative infraocclusion. These habit-induced manifestations typically involve multiple teeth rather than a single isolated tooth [6, 7].

When a tooth remains below the occlusal plane despite a clear eruption pathway, with no associated pathology found and after excluding other potential causes of eruption failure, the tooth is presumed to be ankylosed. Metallic percussion sound was suggested as a clinical test for diagnosing ankylosis. However, this method has not proven to be consistently reliable. Furthermore, it cannot be applied in cases where tooth eruption is arrested prior to emergence into the oral cavity [2, 8, 9].

Radiographic diagnosis of ankylosis depends on its location and size. While proximal root involvement can sometimes be detected on a periapical radiograph, buccal or lingual involvement requires cone beam computed tomography (CBCT). However, small ankylotic areas are easily missed even on a CBCT, meaning a definitive diagnosis is often only confirmed after tooth extraction or when orthodontic forces fail to move the tooth [2, 9, 10].

While ankylosis is a histopathological term, infraocclusion, submergence, mechanical failure of eruption, and primary or secondary retention are used in a clinical context [10].

Various systems for classifying infraoccluded teeth have been described. Brearley et al. have proposed a simplistic classification [11]:

Mild-occlusal surface located approximately 1 mm below the expected occlusal plane of the tooth, moderate-occlusal surface at the level of contact point of one\both adjacent tooth surfaces, severe-occlusal surface at or below the interproximal gingival tissue of one or both adjacent teeth surfaces [11].

Infraocclusion due to ankylosis can affect both deciduous and permanent teeth. In the primary dentition is relatively common, with reported prevalence ranging from 6.6 to 30% [12–15]. In contrast, permanent molar infraocclusion is significantly less frequent, and its prevalence in the literature is limited. As a result, most existing studies on infraocclusion focus on deciduous molars.

Infraoccluded deciduous molars can have several dental implications, affecting both the arch involved and the opposing dentition. These include midline deviation, tipping of the adjacent teeth towards the infraoccluded molar, relative under-eruption of the neighboring teeth, and over-eruption of the opposite molars [16–19]. While deciduous molar infraocclusion is usually self-limiting and often resolves by the natural shedding or extraction of the affected tooth [19–21], the effect of permanent molar Infraocclusion can be long-lasting [3, 8, 22].

Permanent molar infraocclusion can occur at any stage throughout the eruption of the tooth and root development. The effect on dentoalveolar development is progressive with age, it becomes more severe if the eruption stops earlier, as there is still a significant amount of vertical growth expected [22, 23].

In a study investigating the etiology of infraoccluded permanent teeth among a cohort of 12 patients with unilateral retention of two or more teeth, Binner Becktor et al. [3] reported various degrees of dental root malformation, including short roots, taurodontism and hook shaped roots [3]. More recently, Grippaudo et al. [8] investigated permanent first molars eruption failure, encompassing cases of primary failure of eruption, mechanical obstruction and ankylosis. They found that in cases of ankylosis, the affected molars demonstrated dilacerated roots and delayed development compared with the contralateral tooth [8]. However, their group of ankylosed molars was relatively small and included young patients under the age of 9 years before the roots are fully developed.

### Specific objectives or hypotheses

This retrospective two-center study aims to investigate the associated local effects of permanent first molar infraocclusion on root development, specifically morphology and length, in relation to chronological age and the severity of infraocclusion, and the secondary effect of infraocclusion on the position and root development of the adjacent teeth.

## Methods

This retrospective study analyzed panoramic radiographs of 45 patients (aged 9 to 25 years) who were diagnosed with a unilateral infraoccluded permanent first molar.

### Study design

Each patient presented with a single infraoccluded permanent first molar on one side of the jaw, designated as the affected side, while the contralateral molar served as the control side. Affected tooth and adjacent teeth were analyzed for their root development and position in the arch.

### Participants, eligibility criteria, and setting

The sample was selected based on the following inclusion and exclusion criteria:

Inclusion criteria:

- Age between 9 and 25 years
- Presence of a single infraoccluded permanent first molar on one side of the maxilla and/or the mandible.
- Contralateral permanent first molar fully erupted.
- High-quality panoramic radiographs, taken in a 1:1 ratio.

Exclusion criteria:

- Presence of bilateral infraocludded permanent first molars.
- More than one infraoccluded molar within a single quadrant.
- Diagnosis of Primary failure of eruption (PFE).
- Craniofacial syndromes / systemic conditions which might affect tooth eruption
- Functional disturbances
- Poor quality panoramic radiographs unsuitable for accurate measurements.

### Exposures, predictors, potential confounders, and effect modifiers

**Fig. 1:**
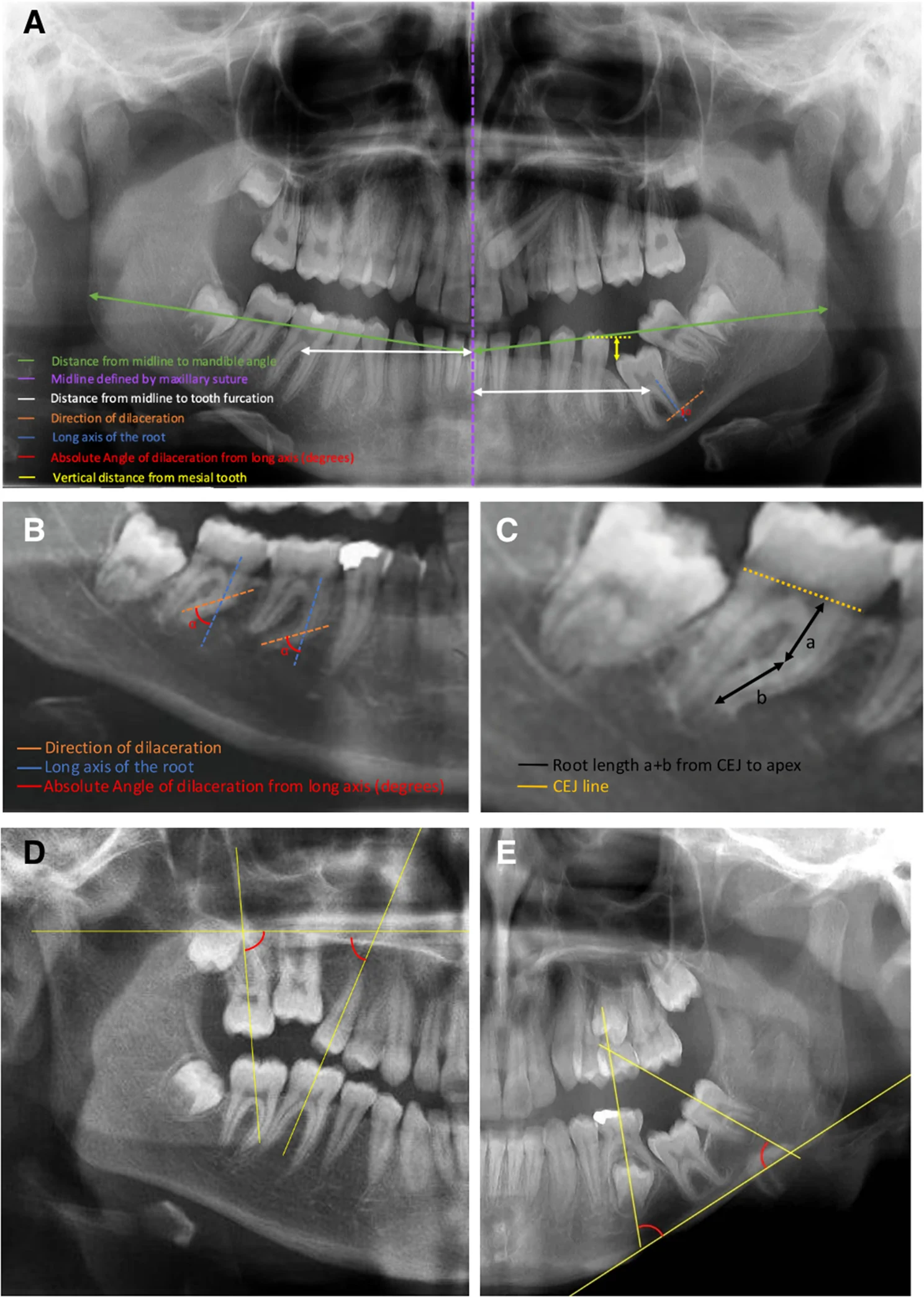
Radiographic measurements used for assessing infraoccluded permanent first molars and adjacent teeth on panoramic radiographs. (**A**) Measurement scheme for tooth position and infraocclusion severity: midline (defined by the maxillary suture/anterior nasal spine), reference distance from midline to mandibular angle, perpendicular distance from midline to tooth furcation (used to generate the tooth-to-midline distance ratio), and vertical infraocclusion severity measured as the distance between the mesial marginal ridge of the infraoccluded molar and the distal marginal ridge of the mesial adjacent tooth. Direction of root dilaceration and the absolute angle of dilaceration (degrees) relative to the long axis are illustrated. (**B**) Dilaceration angle measurement: angle between the long axis of the root and the axis of the dilacerated segment. (**C**) Root length measurement from the cemento-enamel junction (CEJ) line to the apex; in dilacerated roots, total length is calculated as the sum of the straight and curved segments (a+b). (**D**–**E**) adjacent-tooth tipping assessment: long axes of adjacent teeth measured relative to the palatal plane (maxilla) or mandibular plane (mandible)

The exposure variable was whether the permanent first molar (affected side) showed infraocclusion, compared to the contralateral first molar (control side). The severity of infraocclusion was also measured by examining the vertical distance (in millimeters) from the mesial marginal ridge of the infraoccluded tooth to the distal marginal ridge of the mesial adjacent tooth, using Mediadent™ software (Fig. 1A). Potential confounders/effect modifiers included chronological age, sex, affected jaw (maxilla/mandible), and affected side (right/left).

### Outcomes (primary and Secondary,Fig. 1)

1. Dilaceration - In this study, the term ‘root dilaceration’ is used strictly as a descriptive morphological designation to denote a severe curvature or deviation of the root’s longitudinal axis, focusing on the clinical presentation of the root shape regardless of its underlying primary etiology. It was assessed based on angular deviation from the axis of the root (Fig. 1A, B). This measurement was determined by calculating the angle between the root’s long axis and the axis of the dilacerated portion of the root (Fig. 1B). Only angles exceeding 20 degrees were recorded, similar to Chohayeb et al.’s research methodology [24]. This measurement was performed on all examined teeth.
2. Root length - measured in millimeters from the apex to the line connecting both CEJs, using the Mediadent™ software. In dilacerated roots the length was calculated as a sum of the length of the straight and the dilacerated segments (Fig. 1C). Measurements were taken for all teeth examined, including the first and second premolars, and the first and second molars on both the study and control sides.
3. Tooth-to-Midline distance ratio - calculated by measuring the perpendicular distance from the root furcation to the midline, divided by the distance from mandibular angle to the midline at the level of the alveolar crest level (Fig. 1A). the midline is defined by a vertical line along the anterior nasal spine and perpendicular to the palatal plane.
4. Tilting of adjacent teeth - The long axes of each of adjacent teeth were drawn, and the angle of the tilt was measured to the mandibular plane (constructed as a tangent line to the inferior border of the mandible spanning the premolar and gonial angle regions) or the palatal plane (constructed along the clear radiopaque line representing the floor of the nasal cavity/superior surface of the hard palate; Fig. 1D, E)
5. Presence of posterior segment spaces - the existence of spaces between canines, premolars, and molars was recorded in a dichotomous (yes/no) manner.

Primary outcomes were the presence and severity of root dilaceration in the infraoccluded permanent first molar, the differences in root length between the infraoccluded teeth and their corresponding contralateral (unaffected) controls.

Secondary outcomes were adjacent tooth displacement, tipping, posterior spacing, and adjacent tooth root dilaceration. All compared to the control side.

### Sample size calculation

No a priori sample size calculation was performed because this was a retrospective study of consecutively eligible cases of a relatively rare condition available across the participating centers during the study period. In total, 45 infraoccluded molars and their adjacent teeth were assessed on the affected sides, and 45 corresponding molars and adjacent teeth were analyzed on the control side.

### Sources of bias and comparability between exposure groups

To reduce inter-individual variability in craniofacial morphology and developmental timing, a split-mouth design was used, with the contralateral side serving as an internal control. Potential sources of bias included retrospective case selection, reliance on two-dimensional panoramic radiography, and possible misclassification of ankylosis versus other eruption disturbances in younger patients. Comparability between sides was enhanced by within-patient matching, although local developmental asymmetry unrelated to infraocclusion cannot be fully excluded. Panoramic radiography is inherently susceptible to patient positioning variability, leading to nonuniform geometric distortion across the image. Reported evidence indicates that the vertical dimension is comparatively less affected by such positioning errors [25–27]; therefore, direct linear (millimetric) measurements were obtained in the vertical plane. By contrast, the horizontal dimension is particularly vulnerable to magnification and distortion secondary to positioning inaccuracies in panoramic acquisitions [25, 26]. To reduce this source of measurement error, horizontal assessments were based on proportional (ratio-based) measurements rather than absolute linear distances.

### Statistical analysis (primary and secondary outcomes, subgroup analyses, confounding, missing data)

A formal error analysis was conducted to quantify random and systematic errors. A subset of 15 panoramic radiographs was randomly selected from the cohort and remeasured in full by the primary examiner after a three-week interval. Systematic error was evaluated using a paired t-test, which confirmed no statistically significant systematic bias between the evaluation sessions (*p* > 0.05). Random error and measurement reproducibility were quantified using the Intraclass Correlation Coefficient (ICC) for continuous variables and Cohen’s kappa for categorical assessments. Continuous variables showed excellent reproducibility, yielding ICC values of 0.94 for root length and 0.93 for dilaceration angles. Categorical variables also showed high intra-examiner agreement, with a Cohen’s kappa of 0.97.

Most of the analyses were structured according to the split-mouth design of the study, with direct comparisons between the affected side and the contralateral control side within each patient. Continuous paired outcomes, including root length, vertical distance, distance-from-midline ratio, and dilaceration severity (in degrees), were analyzed using paired *t*-tests. These results are presented as between-side differences, together with the corresponding 95% confidence intervals and *p*-values. Dichotomous paired outcomes, including the presence of dilaceration and posterior spacing, were analyzed using McNemar’s test and are reported as paired proportions with the corresponding OR and *p*-values. Effects estimates and 95% confidence intervals are additionally provided in Table 2. Secondary analyses were conducted to examine the association between the type of the adjacent tooth (next to the infraoccluded molar) and the presence of posterior spacing. These analyses were performed using the Chi-square test. No missing data were present for the analyzed variables, as only patients with the required digital radiographic records were included in the study.

A significance level of *p* < 0.05 was assigned to all test findings. These analyses were performed using Prism version 10.2.3, GraphPad Software, Boston, Massachusetts, USA.

To control the inflation of Type I error rates across a wide array of similar dentoalveolar and structural parameters, variables were grouped into predefined logical families of related outcomes, and a post hoc Bonferroni correction was applied within each family. Accordingly, the adjusted significance thresholds were established as follows: (1) Arch Architecture & Spatial Positioning family (6 comparisons, adjusted α = 0.0083); (2) Adjacent Tooth Positional Effects family (4 comparisons, adjusted α = 0.0125); (3) Molar & Adjacent Root Lengths family (6 comparisons, adjusted α = 0.0083); and (4) Root Dilaceration Characteristics & Severity family (13 comparisons, adjusted α = 0.0038).

## Results

### Participant flow

Patient records from the participating centers were retrospectively screened for cases of unilateral infraoccluded permanent first molars among patients who had been accepted for treatment in the departments up to the year 2025 and for whom the required digital radiographic records were available. Because infraocclusion of permanent first molars is a relatively rare condition, the number of eligible cases was inherently limited. Only 5 cases were excluded during screening, all because the patients were younger than the predefined study age range for inclusion. Early stopping was not applicable given the retrospective cross-sectional design. The final study sample, therefore, included 45 patients.

### Baseline data (Table 1)

Patient sample characteristics are presented in Table 1. This study involved 45 orthodontic patients, comprising 24 females and 21 males, aged 9 to 25 years (mean age 12.34 years). All patients presented with a single infraoccluded permanent first molar. Specifically, the infraoccluded molar was located in the maxilla for 14 patients and in the mandible for 31 patients. Furthermore, 19 patients had the infraoccluded molar on the right side, while 26 had it on the left

**Table 1:** Patient characteristics and distribution of infraoccluded permanent first molars. Age summary (mean and range) for the full cohort and by sex, and distribution of infraoccluded molars by jaw (maxilla/mandible) and side (right/left)

Age and Gender		
	**N**	**Mean in years (Range)**
All Patients	45	12.34 (9–25)
Males	21	12.39 (9–25)
Females	24	12.29 (9–23)
**Infraoccluded molar position**		
	**N**	
Maxilla	14	
Mandible	31	
Right	19	
Left	26	

Measurements obtained from the infraoccluded tooth (affected side) were compared with those from the control side.

### Infraoccluded permanent first molars and adjacent teeth position

On the affected side, the permanent first molar was positioned 5.6(3.3)mm lower (*p* < 0.001) than the permanent first molar on the control side.

**Fig. 2:**
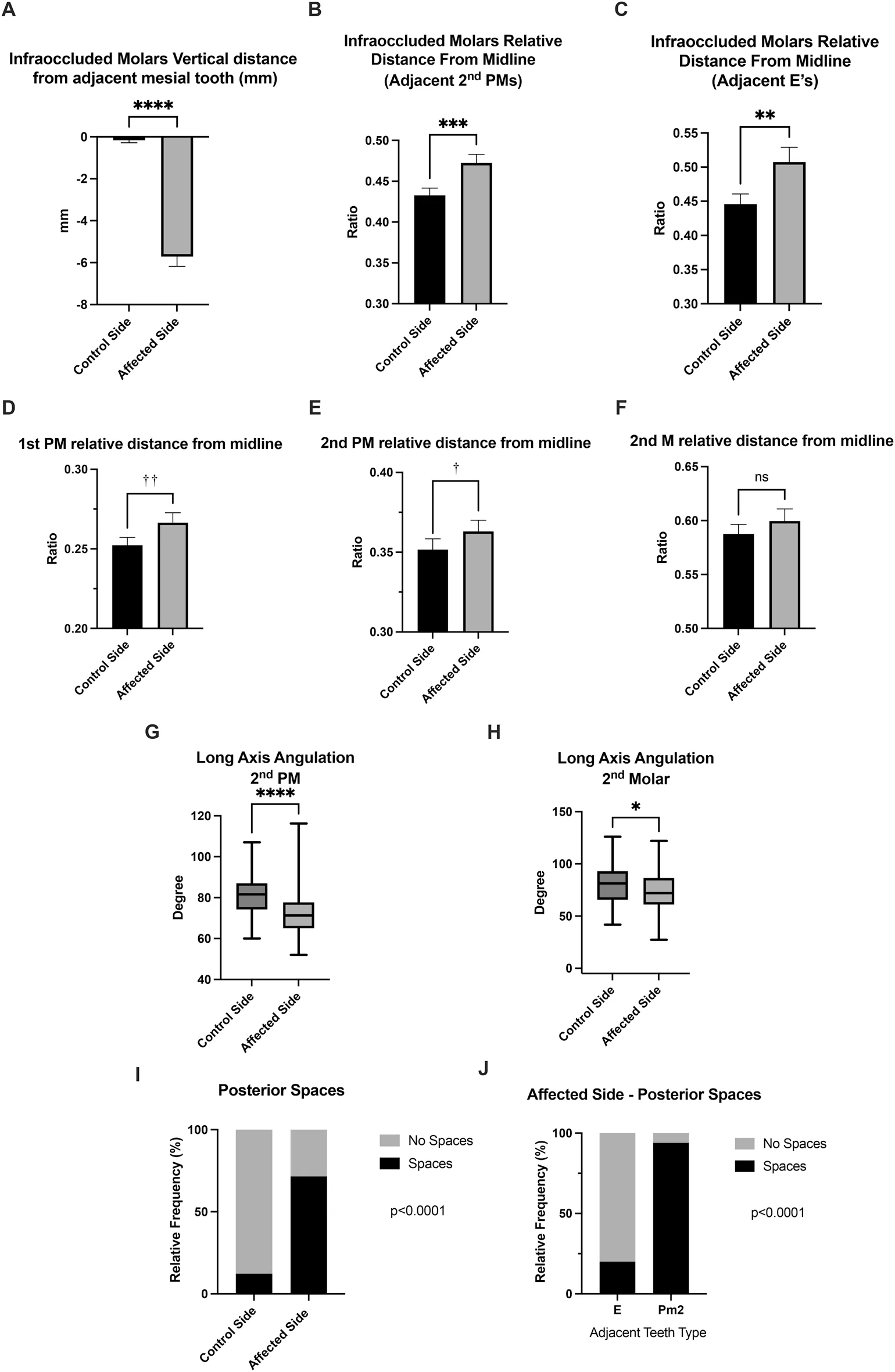
Positional changes of infraoccluded permanent first molars and adjacent teeth (affected vs contralateral control side). (**A**) Severity of infraocclusion expressed as vertical distance (mm) from the infraoccluded permanent first molar to the mesial adjacent tooth. (**B**–**C**) relative distance from the midline (ratio) for infraoccluded permanent first molars, categorized by the mesial adjacent tooth (second premolar vs second primary molar [E]). (**D**–**F**) relative distance from the midline (ratio) for adjacent teeth (first premolar, second premolar, and second molar). (**G**–**H**) long-axis angulation (degrees) of the second premolar and second molar demonstrating tipping toward the infraoccluded molar. (**I**) Relative frequency of posterior segment spacing on the affected versus control side. (**J**) Relative frequency of posterior segment spacing in cases with E vs Pm2 as adjacent to the infraoccluded molars, affected side only. *p* values are shown (*<0.05, **<0.01, ***<0.001, ****<0.0001, ns = non-significant). † denotes nominal statistical significance at the standard unadjusted level (*p* < 0.05); note that this parameter did not maintain significance after the family-wise Bonferroni multiple-comparison correction. * denotes statistical significance remaining after a post-hoc family-wise Bonferroni multiple-comparison correction

The infraoccluded permanent first molar was also found to be positioned significantly more distally compared to the first molar on the control side (*p* < 0.001), regardless of whether the adjacent tooth was the second premolar (PM) or the primary second molar (Es, Fig. 2B,C). Adjacent teeth on the affected side, including the first and second premolars and the second molars, were all shifted more distally, complementing the distal displacement observed in the infraoccluded permanent first molars (*p* < 0.01, *p* = 0.03, *p* < 0.01; Fig. 2D–F), however, the significance of these variations did not survive the strict family-wise Bonferroni multiple-comparison correction. Besides shifting mesio-distally, the adjacent teeth also tilted toward the infraoccluded tooth. Specifically, the second premolars tilted distally, angling 8.98 (12)° degrees more than the controls towards the infraoccluded molar (*p* < 0.001). Likewise, the second molars’ angulation changed, tilting mesially 6.26(19)° degrees more than the control (*p* = 0.003; Fig. 2G, H). Additionally, in 72% of cases on the affected side, there was also significantly more spacing between the posterior teeth compared to only 12% of cases on the unaffected, control side (Matched OR = 15.5;*p* < 0.001; Fig. 2I). We observed significantly more spaces where second premolars were positioned mesially to the infraoccluded molars, but not when second primary molars were present (94% near the Pm2 vs 20% near the E; OR = 62.5;*p* < 0.001, Fig. 2J).

### Root development of the infraoccluded molars

**Fig. 3:**
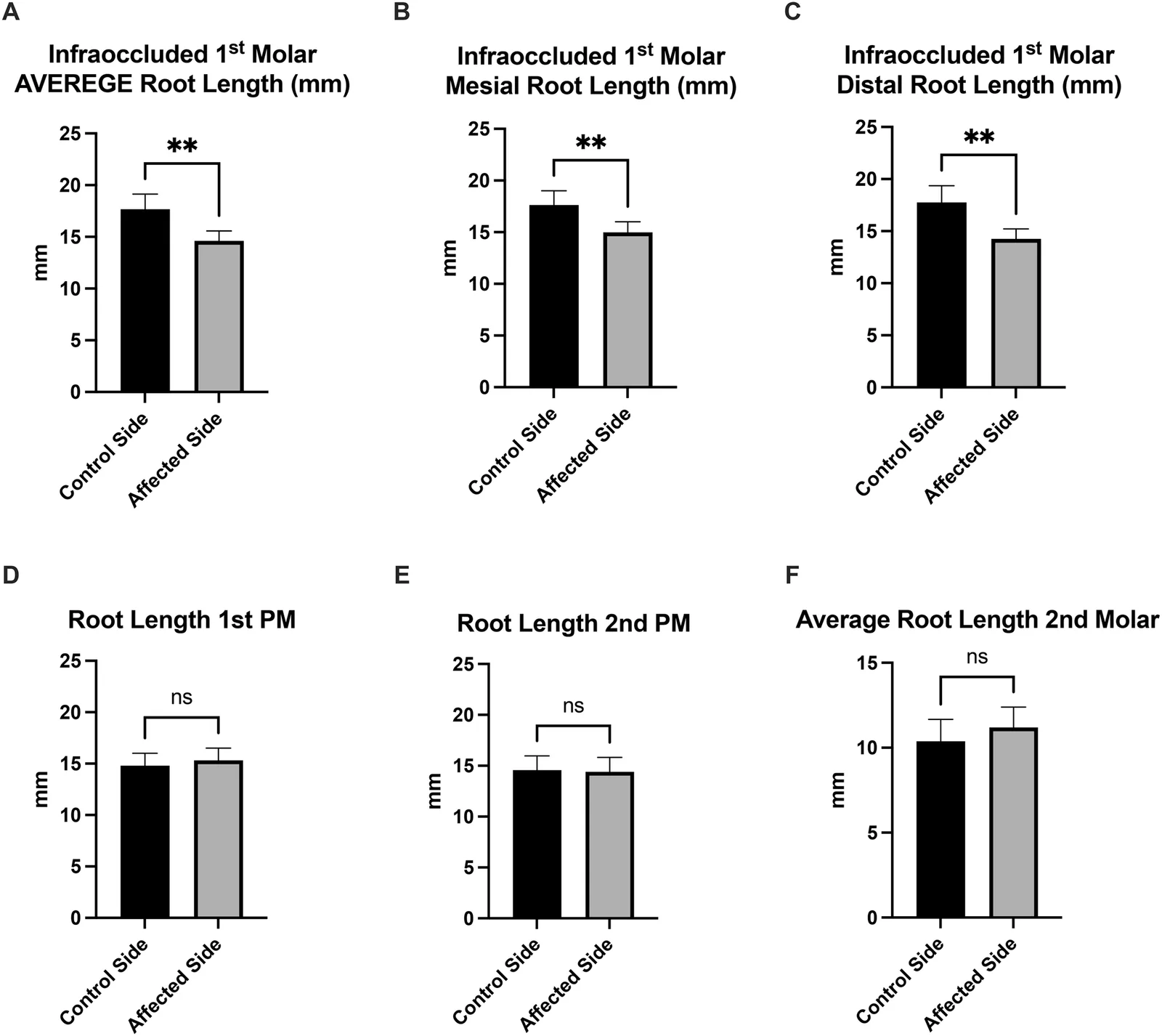
Root length comparisons of infraoccluded permanent first molars and adjacent teeth on affected vs control sides. (**A**) Average root length (mm) of infraoccluded permanent first molars compared with contralateral controls. (**B**–**C**) mesial and distal root lengths (mm) of the permanent first molar. (**D**–**F**) root lengths (mm) of adjacent teeth (first premolar, second premolar, and second molar). *p* values are shown (*<0.05, **<0.01, ***<0.001, ****<0.0001, ns = non-significant). † denotes nominal statistical significance at the standard unadjusted level (*p* < 0.05); note that this parameter did not maintain significance after the family-wise Bonferroni multiple-comparison correction. * denotes statistical significance remaining after a post-hoc family-wise Bonferroni multiple-comparison correction

**Fig. 4:**
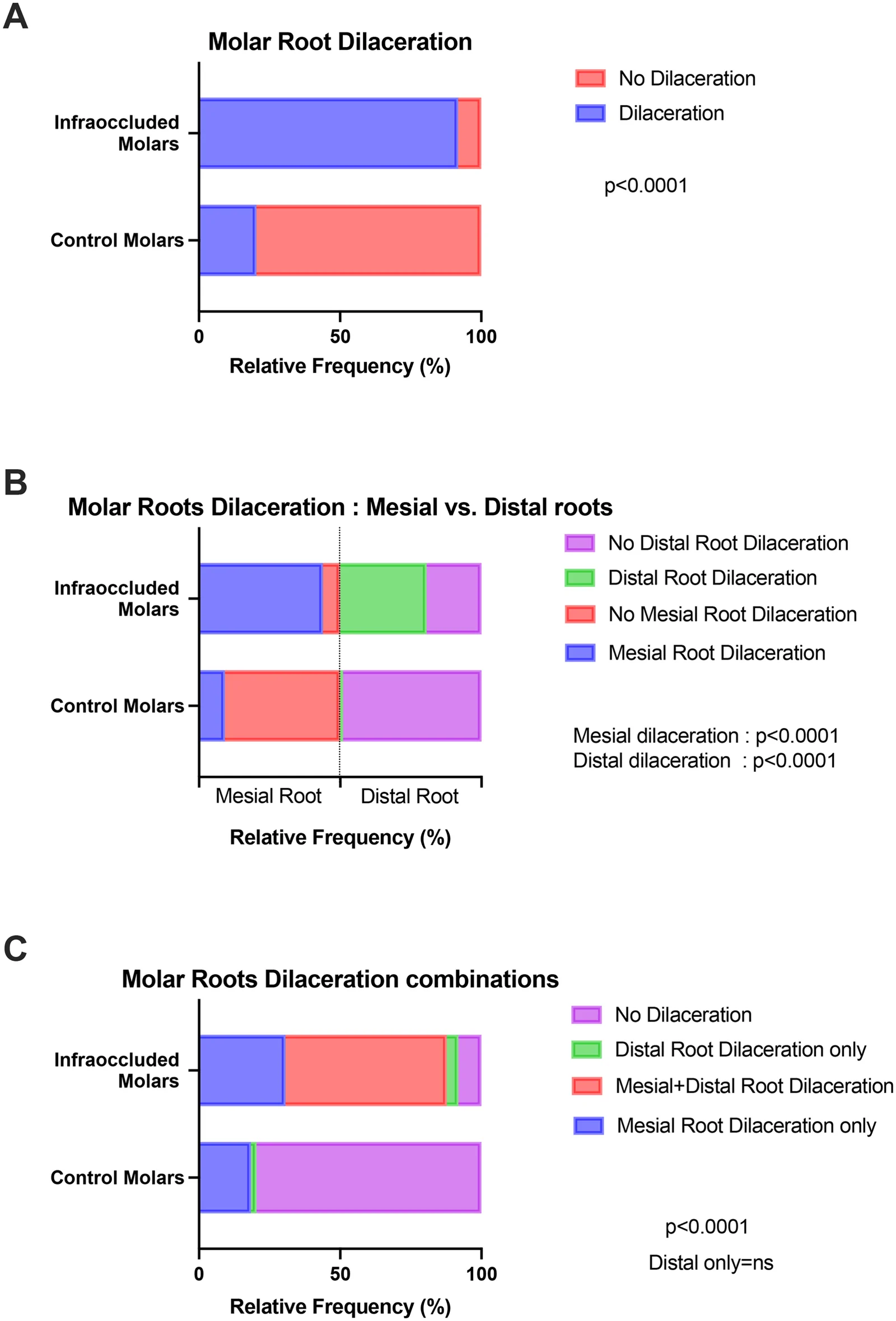
Prevalence and distribution of root dilaceration in infraoccluded permanent first molars versus contralateral controls. (**A**) Presence/absence of any root dilaceration (percentage). (**B**) Distribution of dilaceration by root (mesial vs distal) in permanent first molars. (**C**) Dilaceration pattern combinations (mesial only, distal only, both roots, or none)

The infraoccluded permanent first molars had significantly shorter average root lengths compared to the control permanent first molars with a mean difference of −2.8 (5.4)mm (*p* < 0.001; Fig. 3A). Similar significant reductions were seen when analyzing the mesial and distal root lengths individually [−2.5(4.9)mm, *p* < 0.001, and −3.38 (6.4)mm, *p* < 0.001, respectively; Fig. 3B,C]. However, the adjacent teeth did not exhibit a comparable influence on root length (Fig. 3D,E,F), and no notable differences were observed between the groups. The roots of the infraoccluded permanent first molars were not only shorter but were found to have significantly more dilaceration compared to the control permanent first molars. We observed that 92% of the infraoccluded permanent first molars exhibited any kind of root dilaceration compared to only 21% of the permanent first molars on the control side (Matched OR = 18.5;*p* < 0.001) (Fig. 4A). Specifically, 88% of the infraoccluded permanent first molars presented mesial root dilaceration compared to only 18% on the control side (Matched OR = 18;*p* < 0.001). In addition, distal root dilaceration was found in 62% of infraoccluded permanent first molars, with only 11% of distal root dilaceration detected on the control side (Matched OR = 15;*p* = 0.001) (Fig. 4B).

We also investigated the co-occurrence of dilaceration in both the mesial and distal roots. On the affected side, 31% of the infraoccluded permanent first molars showed dilaceration in the mesial root only, 57% had dilaceration in both mesial and distal roots, 4% showed dilaceration in the distal root only, and 8% had no root dilaceration (Fig. 4C). In contrast, on the control side, 19% of the permanent first molars exhibited mesial root dilaceration alone, 11% had only distal root dilaceration, and the remaining 70% showed no root dilaceration. The differences between the groups were highly significant (Fig. 4C, *p* < 0.001).

**Fig. 5:**
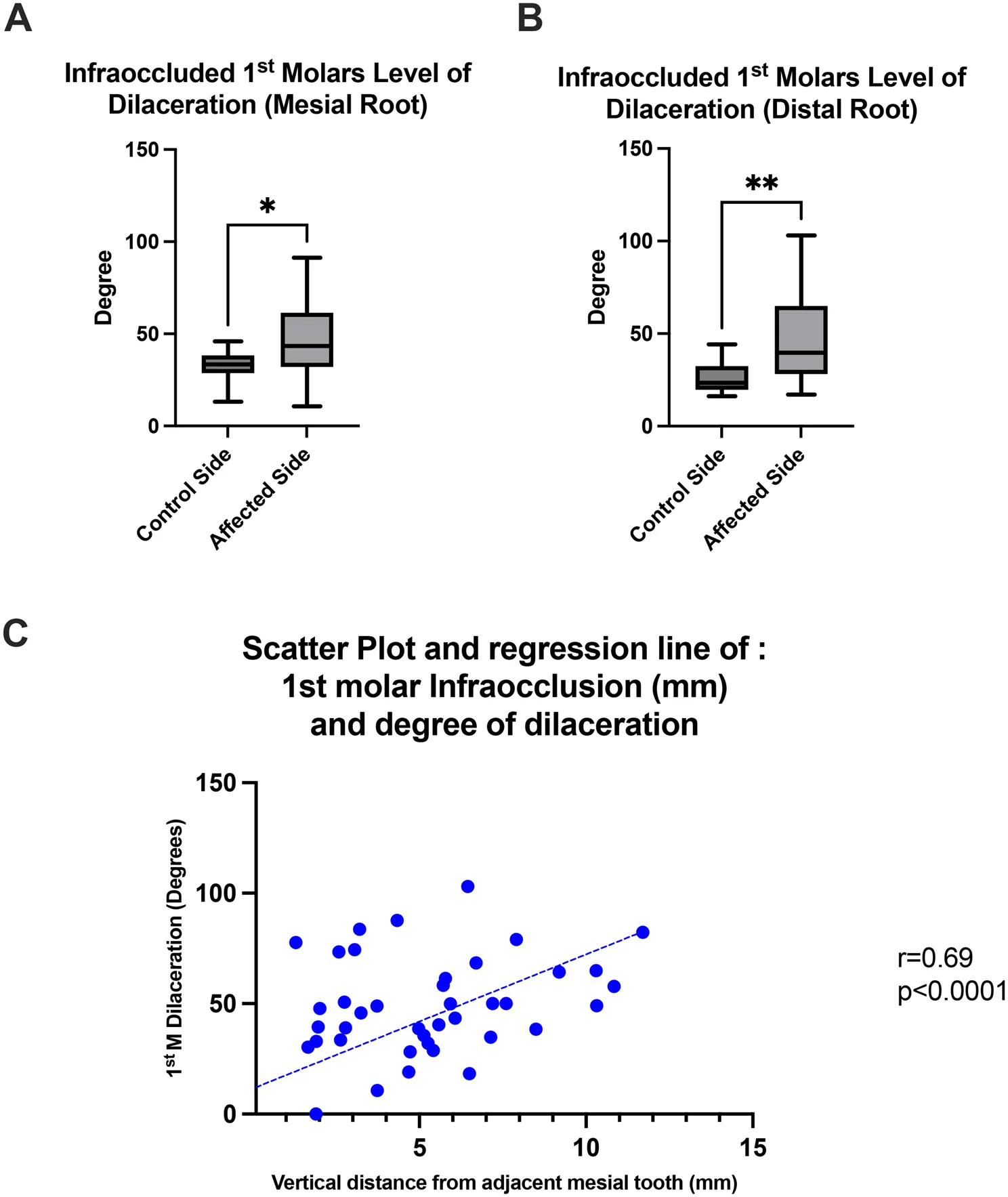
Severity of dilaceration in infraoccluded permanent first molars and its association with infraocclusion severity. (**A**) Dilaceration angle (degrees) in the mesial root of the permanent first molar (affected vs control side). (**B**) Dilaceration angle (degrees) in the distal root (affected vs control side). (**C**) Scatter plot with regression line depicting the association between infraocclusion severity (vertical distance, mm) and dilaceration severity (degrees) for infraoccluded permanent first molars. *p* values are shown (*<0.05, **<0.01, ***<0.001, ****<0.0001, ns = non-significant). † denotes nominal statistical significance at the standard unadjusted level (*p* < 0.05); note that this parameter did not maintain significance after the family-wise Bonferroni multiple-comparison correction. * denotes statistical significance remaining after a post-hoc family-wise Bonferroni multiple-comparison correction

To assess the severity of the dilaceration, we measured the angle of curvature in the mesial and distal roots of the infraoccluded permanent first molars. We found significantly more severe dilaceration in the infraoccluded permanent first molars compared to the control side, affecting both the mesial (47° vs. 32°; *p* = 0.002) and distal roots (47° vs. 26°; *p* = 0.0015) (Fig. 5A,B).

Finally, we examined the relationship between the severity of dilaceration and the extent of infraocclusion. Using scatter plot visualization alongside linear regression analysis, we identified a statistically significant linear association between the severity of infraocclusion and the severity of dilaceration, with a coefficient of determination R^2^ = 0.5, Spearman correlation coefficient *r* = 0.69 (*p* < 0.001, Fig. 5C).

### Effect on adjacent teeth development

**Fig. 6:**
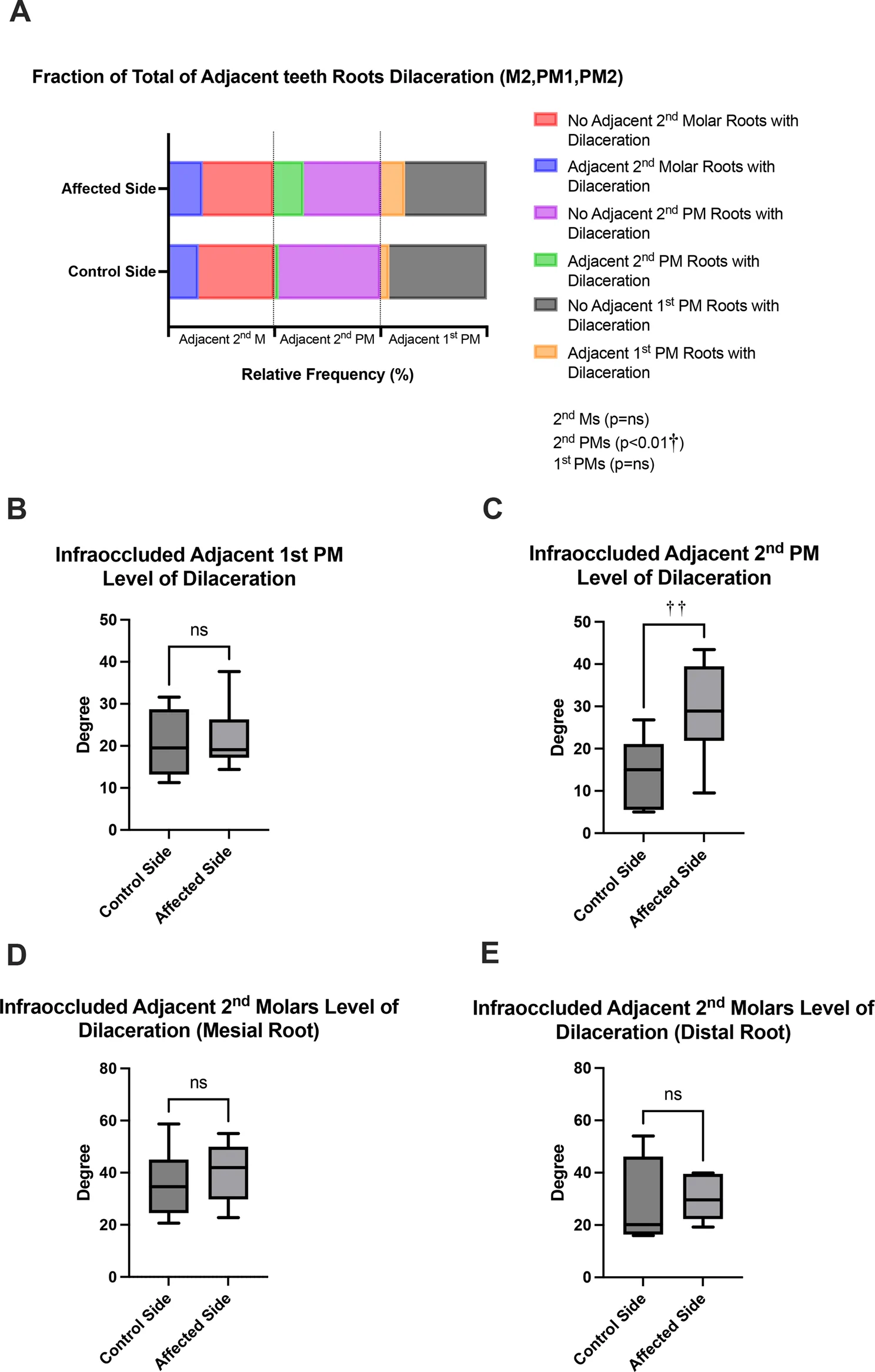
Effect of infraoccluded permanent first molars on root dilaceration in adjacent teeth. (**A**) Relative frequency of dilaceration (percentage) in roots of adjacent teeth (second molar, second premolar, first premolar) on the affected versus control side. (B–C) dilaceration angle (degrees) in the first premolar and second premolar. (D–E) dilaceration angle (degrees) in mesial and distal roots of the adjacent second molar. *p* values are shown (*<0.05, **<0.01, ***<0.001, ****<0.0001, ns = non-significant). † denotes nominal statistical significance at the standard unadjusted level (*p* < 0.05); note that this parameter did not maintain significance after the family-wise Bonferroni multiple-comparison correction. * denotes statistical significance remaining after a post-hoc family-wise Bonferroni multiple-comparison correction

We also investigated how infraoccluded permanent first molars affected the presence of dilaceration in adjacent teeth. The prevalence of dilaceration tended to be higher on the affected side in all adjacent teeth. Specifically, the first premolars showed dilaceration in 24% of cases versus 8% on the control side (Matched OR = 3.33;*p* = ns), the second premolars in 28% versus 4% (Matched OR = 13;*p* = 0.0012), and the second molars in 32% versus 28% (Matched OR = 2.5;*p* = ns) (Fig. 6A). The root dilaceration severity was also assessed in the teeth adjacent to the infraoccluded permanent first molars. The first premolars and second molars showed no significant difference between the affected and control sides (Fig. 6B, D, E). However, the second premolar on the affected side exhibited significantly more severe dilaceration compared to the unaffected side (30° vs 13°, *p* < 0.01, Fig. 6C).

It is important to note that the significance of the adjacent teeth development variations did not survive the strict family-wise Bonferroni multiple-comparison correction.

### The effects of chronological age, sex, affected jaw, and affected side

The analyses examining the associations between these demographic parameters and the primary and secondary outcomes demonstrated only weak associations. Specifically, chronological age showed a weak correlation with the severity of infraocclusion on the affected side (*r* = 0.40).

### Numbers analyzed for each outcome, estimation and precision

The number of observations analyzed for each outcome is shown in Table 2. Most of the main analyses were based on within-patient paired comparisons between the infraoccluded and contralateral sides, except for the assessment of dilaceration severity, for which only teeth with dilaceration were included. Accordingly, the reported results reflect the magnitude and direction of differences between sides for dentoalveolar position, root length, and root developmental changes. When applicable, the statistical results are presented alongside their corresponding *p*-values.

**Table 2:** Numbers analyzed, effect estimates, and statistical precision for the principal analyses. The table presents the main comparisons between the affected side, containing the infraoccluded permanent first molar, and the contralateral control side. Continuous outcomes are expressed as mean (SD), and the corresponding between-side differences are shown with 95% confidence intervals and *p*-values. Dichotomous outcomes are presented as percentages. in keeping with the split-mouth design, most comparisons were performed as paired within-patient analyses. for dilaceration severity, only teeth exhibiting dilaceration were included; therefore, the number of analyzed observations for these variables differs from the full study sample. Reduced sample sizes in selected rows also reflect subgroup analyses based on the type of mesial adjacent tooth

Outcome	n analyzed	Control side	Affected side	Effect estimate	95% CI	p-value
Infraocclusion severity of 1st molar (vertical distance from mesial adjacent tooth, mm)	45 pairs	0 mm	−5.6 (3.3)mm	Mean paired difference: −5.6(3.3)mm	−6.6 to −4.6	<0.001^*^
Relative distance from midline of infraoccluded 1st molar, adjacent 2nd premolar subgroup	33 pairs	0.43(0.05)	0.47(0.06)	Mean paired difference in ratio: 0.04(0.05)	0.02 to 0.06	<0.001^*^
Relative distance from midline of infraoccluded 1st molar, adjacent second primary molar (E) subgroup	12 pairs	0.44(0.05)	0.50(0.08)	Mean paired difference in ratio : 0.06(0.07)	0.02 to 0.1	<0.01^*^
Relative distance from midline of adjacent 1st premolar	45 pairs	0.25(0.03)	0.27(0.04)	Between-side difference in ratio: 0.014(0.03)	0.004 to 0.023	<0.01^†^
Relative distance from midline of adjacent 2nd premolar	33 pairs	0.35(0.04)	0.36(0.04)	Between-side difference in ratio: 0.011(0.04)	0.00 to 0.02	0.03^†^
Relative distance from midline of adjacent 2nd molar	45 pairs	0.58(0.05)	0.6(0.07)	Between-side difference in ratio:0.011(0.06)	0.00 to 0.03	<0.01^†^
Long-axis angulation of adjacent 2nd premolar	33 pairs	81.96(10.26)°	73.6(13)°	Mean paired difference: −8.38(12)°	−11.9 to −4.8	<0.001^*^
Long-axis angulation of adjacent 2nd molar	45 pairs	80.21(17)°	73.9(19)°	Mean paired difference: −6.26(19)°	−11.89 to −0.64	<0.01^*^
Posterior spacing presence	45 pairs	12%	72%	Matched OR = 15.50	3.94 to 133.67	<0.001^*^
Posterior spacing – **Affected side only** (Adjacent E vs Pm2)	16 E, 29 Pm2		20% spacing (E), 94% spacing (Pm2)	OR = 62.5	81.3 To 434	<0.001^*^
Average root length of infraoccluded 1st molar	45 pairs	17.7(9.7)mm	14.6(6.6)mm	Mean paired difference: −2.8(5.4) mm	−4.4 to −1.2	<0.001^*^
Mesial root length of infraoccluded 1st molar	45 pairs	17.65(9.1)mm	14.98(6.9)mm	Mean paired difference: −2.51(4.9)mm	−4 to −1	<0.001^*^
Distal root length of infraoccluded 1st molar	45 pairs	17.75(10.6)mm	14.25(6.55)mm	−3.38(6.4)mm	−5.34 to −1.43	<0.001^*^
Root length of adjacent 1st premolar	45 pairs	14.8(7.9)mm	15.3(8)mm	Mean paired difference: 1.16(4)mm	−0.15 to 2.4	0.08
Root length of adjacent 2nd premolar	33 pairs	14.58(8.7)mm	14.4(9.1)mm	Mean paired difference: 0.098(3.51)mm	−1.108 to 1.27	0.86
Root length of adjacent 2nd molar	45 pairs	10.38(8)mm	11.19(7.5)mm	Mean paired difference: 0.355(3)mm	−0.7 to 1.41	0.49
Presence of any root dilaceration in 1st molar	45 pairs	21%	92%	Matched OR = 18.50	4.77 to 158.44	<0.001^*^
Presence of mesial root dilaceration in 1st molar	45 pairs	19%	88%	Matched OR = 18	4.6 to 154.3	<0.001^*^
Presence of distal root dilaceration in 1st molar	45 pairs	11%	62%	Matched OR = 15	3.8 to 129.5	<0.001^*^
Mesial-root dilaceration severity in 1st molar	8 Control, 40 Affected	32°	47°	Mean difference: 15(4.4)°	6.4 to 24.47	<0.01^*^
Distal-root dilaceration severity in 1st molar	5Control, 28 Affected	26°	47°	Mean difference: 21(4.9)°	11.15 to 31.29	<0.01^*^
Association between infraocclusion severity and 1st molar dilaceration severity	45 pairs	—	—	r = 0.69	0.49 to 0.76	<0.001^*^
Presence of dilaceration in adjacent 1st premolar	45 pairs	8%	24%	Matched OR = 3.33	0.85 to 18.84	0.09
Presence of dilaceration in adjacent 2nd premolar	45 pairs	4%	28%	Matched OR = 13	1.95 to 552	<0.01^†^
Presence of dilaceration in adjacent 2nd molar	45 pairs	28%	32%	Matched OR = 2.5	0.72 to 10.92	0.18
Dilaceration severity in adjacent 1st premolar	4 Control, 11 Affected	20.48(8)°	22.6(7)°	Mean paired difference: 1.3(8) °	−10.2 to 14	0.67
Dilaceration severity in adjacent 2nd premolar	5 Control, 14 Affected	13.6(8.8)°	29.7(10.3)°	Mean difference: 16.1(9)°	5.06 to 27.1	<0.01^†^
Dilaceration severity in adjacent 2nd molar, mesial root	11 Control, 14 Affected	35.35(11)°	40.51(10)°	Mean paired difference:5.15(10)°	−4.1 to 14.44	0.26
Dilaceration severity in adjacent 2nd molar, distal root	4 Control, 6 Affected	27.5(17)°	30.6 (9)°	Mean paired difference:3.07(9)°	−23.58 to 29	0.76

†Statistically significant at the standard unadjusted level (*p* < 0.05)

* Remains statistically significant after applying a family-wise Bonferroni correction within logical categories of related outcomes: • Arch Architecture & Spatial Positioning family (threshold *p* < 0.0083; includes: infraocclusion severity, relative distance from midline of 1st molar for both 2nd premolar and 2nd primary molar subgroups, and relative distance from midline for adjacent 1st premolar, 2nd premolar, and 2nd molar). • Adjacent Tooth Positional Effects family (threshold *p* < 0.0125; includes: long-axis angulation of adjacent 2nd premolar, long-axis angulation of adjacent 2nd molar, posterior spacing presence, and adjacent E vs Pm2 spacing subgroup). • Molar & Adjacent Root Lengths family (threshold *p* < 0.0083; includes: average, mesial, and distal root lengths of the infraoccluded 1st molar, and root lengths of the adjacent 1st premolar, 2nd premolar, and 2nd molar)

• Root Dilaceration Characteristics & Severity family (threshold *p <* 0.0038; includes: presence of any, mesial, or distal root dilaceration in the 1st molar; mesial and distal root dilaceration severity in the 1st molar; association between infraocclusion and 1st molar dilaceration severity; presence of dilaceration in adjacent 1st premolar, 2nd premolar, and 2nd molar; and dilaceration severity in adjacent 1st premolar, 2nd premolar, and 2nd molar mesial/distal roots).

## Discussion

The aim of this study was to investigate the impact of infraoccluded permanent first molars on the development of the affected tooth as well as on the adjacent teeth.

This two-center study included a substantial sample size and used a split-mouth design, providing reliable intraindividual contralateral control teeth and minimizing interindividual variability. In contrast to previous studies, which often involved smaller cohorts and lacked control groups, our study design strengthens the validity and generalizability of the findings.

### Main findings in the context of the existing evidence, interpretation

#### Size and morphology of the Infraoccluded permanent first molar roots

The findings demonstrate a significant association between ankylosed molars and both reduced root length and the presence of root dilacerations. Infraoccluded molars exhibited a markedly higher frequency of root dilacerations compared with controls. The prevalence of molar root dilacerations reported in the literature ranges from 1 to 29% [28–30], depending on the population studied and diagnostic criteria. Most previous investigations have defined dilaceration as a deviation exceeding 90 degrees, whereas in the present study, dilacerations greater than 20 degrees were registered [24]. Consequently, the prevalence observed in our control group was higher than that reported in the literature, reflecting the difference in measurement thresholds. Notably, the prevalence in the affected teeth was approximately 4 times higher than in the control teeth. The vast majority of infraoccluded permanent first molars demonstrated some degree of root dilaceration, emphasizing the strong association between infraocclusion and altered root morphology.

Both curvature and the number of affected roots were significantly greater among infraoccluded molars. Asheghi B et al. reported that mesial root dilaceration is the most frequently observed type in normally developing molars [30]. In contrast, our findings revealed an increased prevalence of both mesial and distal root dilaceration in infraoccluded teeth. This suggests that the severity of dilaceration is not solely determined by the degree of curvature, but also by the number of affected roots. Furthermore, the presence of dilaceration at the distal root may serve as a warning indicator for potential eruption disturbances.

### The extent of infraocclusion and the Impact on Root Dilaceration

A direct relationship was observed between the extent of infraocclusion and the severity of root dilaceration (Fig. 5C). This finding supports our hypothesis that infraoccluded teeth continue root development while the vertical space available for this process becomes progressively restricted by the cortical bone or periosteum of anatomical structures, potentially impairing normal root formation. Similar patterns have been observed in impacted teeth, supporting the notion that spatial restriction during tooth development contributed to root deformities [31, 32].

### Distal positioning and dental asymmetry

The infraoccluded permanent first molar and its adjacent teeth were found to be positioned more distally within the dental arch compared with their counterparts in the control teeth. Moreover, adjacent teeth exhibited significant tilting towards the infraoccluded tooth, with the second premolars and second molars displaying excessive tipping compared with controls. These observations are consistent with previous findings by Becker et al. [16, 18], who reported that infraoccluded primary molars tend to occupy a more distal position relative to controls and a tilting of adjacent teeth toward the infraoccluded deciduous molar. It has been suggested that these effects are mediated through the transseptal fibers, which exert a pulling force toward the infraoccluded tooth [16, 18]. To the best of our knowledge, the present study is the first to demonstrate these dental effects specifically in relation to permanent infraoccluded first molars.

The distal positioning of the second molar associated with an infraoccluded permanent first molar may further contribute to third molar impaction. A reduction in the space between the distal surface of the second molar and the anterior border of the mandibular ramus has been shown to increase the likelihood of impaction, whereas mesial migration of the second molar reduces this risk [33].

In addition, the affected side displayed considerably greater spaces compared with the control side. The spaces on the affected side, were highly correlated when the adjacent tooth was the second premolar, but not when the adjacent tooth was the primary second molar. This might be explained by the persistence of the leeway space, which can not close naturally due to lack of physiologic mesial drift [18].

### Root morphology of the adjacent teeth

A significant increase in the extent of root dilaceration was observed, primarily in the adjacent premolars. To the best of our knowledge, this study is the first to demonstrate root morphological alterations in teeth adjacent to infraoccluded permanent first molars. These findings suggest that the adjacent teeth are subjected to developmental constraints imposed by the infraoccluded tooth, likely mediated through the transseptal fibers that restrict normal eruptive and mesial drift movements of the adjacent teeth, thereby contributing to the observed root dilacerations and their increased severity.

### Clinical relevance

The alterations in teeth position: the distal position of the teeth on the affected side and the angulation of the adjacent teeth to the infraoccluded molars may lead to dental asymmetry in unilateral cases, manifested as an asymmetric buccal relationship. Additionally, facial asymmetry and occlusal canting have been reported in association with unilateral infraocclusion [22], suggesting that localized infraocclusion may have wider implications for craniofacial symmetry and occlusal development. Moreover, we saw a reduction in root length of the affected teeth by approximately 15%. Reduced root length decreases effective root support and worsens the crown-root ratio, which may have an effect later on if a periodontal condition also reduces the bone support of that tooth. Likewise, root dilaceration is not merely a radiographic feature. It may complicate orthodontic mechanics [34] and can make future endodontic treatment more difficult, as diagnosis, access, canal preparation, and obturation are all reported to be more challenging in dilacerated teeth, and the prognosis of these procedures decreases as the severity of the dilaceration increases [35].

### Challenges in Differential Diagnosis

The diagnosis of an ankylosed infraoccluded molar can be challenging. Among the challenges is the differentiation from Primary Failure of Eruption (PFE) [36]. Both conditions may present overlapping clinical and radiographic characteristics, particularly at an early dental age, prior to the eruption of all permanent molars. Grippaudo et al. proposed that the presence of root dilacerations could serve as a useful differentiating feature, as dilacerations were more frequently observed in ankylosed teeth compared with teeth affected by PFE. Our findings support the notion that infraoccluded ankylosed teeth display a higher frequency of dilaceration compared with controls. Thereby, the presence of a dilacerated infraoccluded molar may indicate an ankylosis rather than PFE [8].

### Intervention timing and treatment options

As is widely recognized, infraocclusion becomes increasingly severe over time, especially if the ankylosis occurred at an early age. Thus, the timing of intervention can be crucial in the management of infraoccluded ankylosed molars. Previous studies have demonstrated that the earlier a tooth becomes ankylosed, the greater the degree of infraocclusion that develops [16–18, 37]. Accordingly, early ankylosis may exert a greater influence on subsequent tooth development, the development and position of adjacent teeth, and overall dental asymmetry. Early diagnosis allows for preventive measures that help prevent adverse effects on the developing dentition and minimize both dental and skeletal asymmetry.

Treatment options include luxation and orthodontic traction of the affected tooth or, alternatively, early extraction to facilitate eruption of the second molar into the extraction space [23, 38]. Delayed diagnosis may necessitate more complex surgical or orthodontic procedures, often associated with less favorable outcomes. In both approaches, the procedure is generally less complicated if the tooth roots are not dilacerated. Straight roots allow for more predictable eruption or traction, whereas extraction is ideally performed before full root formation to avoid complications. Dilacerated roots, often in close proximity to vital structures such as the inferior alveolar canal or maxillary sinus due to submergence, significantly increase surgical complexity. Extraction of ankylosed teeth with root dilaceration is particularly challenging, as it carries an increased risk of root fracture or damage to adjacent anatomical structures. Similarly, orthodontic traction of a dilacerated tooth is difficult to achieve and is associated with a higher risk of root resorption, alveolar bone loss, and injury to neighboring structures during attempted movement. The presence of dental asymmetry further complicates treatment planning and execution.

### Limitations

1. The study cohort consisted exclusively of patients referred to specialized orthodontic clinics. Thus, the retrospective nature and sampling framework of this study introduce an inherent referral bias.
2. In younger patients, diagnosing ankylosed infraoccluded permanent first molars is particularly challenging, as it is difficult to definitively distinguish between delayed eruption and PFE at early stages. Between the ages of 9 and 11, diagnosis remains difficult, whereas by around 11–12 years, when the development and eruption status of all the first and second permanent molars are clearer, the diagnosis becomes more straightforward. Thus, patients below the age of 9 were not included in this study.
3. The reliance on two-dimensional panoramic images to evaluate tooth and root morphology. Although CBCT would allow for a more precise and comprehensive evaluation, in the present study it was essential to assess both sides of the dentition, and priority was given to minimizing radiation exposure in growing children.
4. The split-mouth design provides an intraindividual reference and inherently controls for chronological age, sex, genetic background, and systemic growth-related factors. Nevertheless, this design has inherent limitations. Chronological age cannot confound the within-patient comparison between the affected and contralateral sides, as both sides are evaluated at the same age. However, because the exact timing of ankylosis onset cannot be determined retrospectively, age and the remaining growth potential may modify the magnitude of the observed asymmetry, since the consequences of arrested eruption may accumulate during continued dentoalveolar growth. In addition, the clinically unaffected contralateral side should be regarded as an intraindividual reference rather than an independent normal biological control [39]. Although patients with PFE and other syndromic or systemic conditions known to affect tooth eruption were excluded, both sides necessarily share the same genetic background and may share other biological susceptibilities affecting dental development. Therefore, the possibility of subclinical alterations on the contralateral side cannot be excluded.

*Generalizability:*

The findings of this two-center retrospective split-mouth study are likely most applicable to patients with unilateral infraocclusion of permanent first molars treated in specialized orthodontic settings and assessed with digital panoramic radiographs. The two-center approach enhances broader clinical relevance; however, caution should be exercised when applying these results to bilateral cases, syndromic patients, or populations evaluated with different imaging techniques. Additionally, since the sample was taken from patients referred for treatment to specialist departments, the results may better reflect referral-based clinical populations than the general population.

## Conclusions

Unilateral infraocclusion of a permanent first molar was associated with a distinct pattern of dentoalveolar disturbance:

- The affected molar and adjacent teeth were positioned more distally within the arch. Adjacent teeth tipped toward the infraoccluded tooth and posterior spacing was substantially more frequent on the affected side, collectively predisposing to unilateral occlusal asymmetry.
- The infraoccluded molar demonstrated impaired root development, characterized by shorter roots and a markedly higher prevalence and severity of root dilaceration, with a significant positive association between infraocclusion severity and dilaceration severity.
- Developmental effects extended beyond the affected molar, as adjacent teeth, particularly second premolars, showed increased prevalence and, in some cases, greater severity of dilaceration on the affected side, suggesting a broader local effect on posterior dental development.
- The observed positive correlations between infraocclusion severity and dilaceration severity indicate that root deformity may become more pronounced with time in untreated cases.

These findings highlight the importance of early identification and timely intervention for infraoccluded permanent first molars. This proactive approach can help prevent further root deformities and reduce the progression of asymmetry, ultimately simplifying future treatment.

## Data Availability

The datasets used and/or analyzed during the current study are available from the corresponding author on reasonable request.

## Declarations

### Registration and protocol accessibility

As this was a retrospective cross-sectional study, no prospective registration was performed, and no publicly accessible study protocol was available.
